# High Temperature and Elevated Carbon Dioxide Modify Berry Composition of Different Clones of Grapevine (*Vitis vinifera* L.) cv. Tempranillo

**DOI:** 10.3389/fpls.2020.603687

**Published:** 2020-12-01

**Authors:** Marta Arrizabalaga-Arriazu, Eric Gomès, Fermín Morales, Juan José Irigoyen, Inmaculada Pascual, Ghislaine Hilbert

**Affiliations:** ^1^Universidad de Navarra, Faculty of Sciences, Plant Stress Physiology Group, Associated Unit to CSIC (EEAD, Zaragoza, and ICVV, Logroño), Pamplona, Spain; ^2^EGFV, Univ. Bordeaux, Bordeaux Sciences Agro, INRAE, ISVV, Villenave d’Ornon, France; ^3^Instituto de Agrobiotecnología (IdAB), Consejo Superior de Investigaciones Científicas (CSIC)-Gobierno de Navarra, Pamplona, Spain

**Keywords:** climate change, grapevine (*Vitis vinifera*), genetic variability, sugars, malic acid, amino acids, anthocyanins

## Abstract

Tempranillo is a grapevine (*Vitis vinifera* L.) variety extensively used for world wine production which is expected to be affected by environmental parameters modified by ongoing global climate changes, i.e., increases in average air temperature and rise of atmospheric CO_2_ levels. Apart from determining their effects on grape development and biochemical characteristics, this paper considers the intravarietal diversity of the cultivar Tempranillo as a tool to develop future adaptive strategies to face the impact of climate change on grapevine. Fruit-bearing cuttings of five clones (RJ43, CL306, T3, VN31, and 1084) were grown in temperature gradient greenhouses (TGGs), from fruit set to maturity, under two temperature regimes (ambient temperature vs. ambient temperature plus 4°C) and two CO_2_ levels (ambient, ca. 400 ppm, vs. elevated, 700 ppm). Treatments were applied separately or in combination. The analyses carried out included berry phenological development, the evolution in the concentration of must compounds (organic acids, sugars, and amino acids), and total skin anthocyanins. Elevated temperature hastened berry ripening, sugar accumulation, and malic acid breakdown, especially when combined with high CO_2_. Climate change conditions reduced the amino acid content 2 weeks after mid-veraison and seemed to delay amino acidic maturity. Elevated CO_2_ reduced the decoupling effect of temperature on the anthocyanin to sugar ratio. The impact of these factors, taken individually or combined, was dependent on the clone analyzed, thus indicating certain intravarietal variability in the response of Tempranillo to these climate change-related factors.

## Introduction

Grapevine is one of the most prominent crops in agriculture given the cultural and economic importance of grape and wine production. Among the grape varieties cultivated worldwide, Tempranillo ranked #3 in 2017 with 231,000 ha, behind Cabernet Sauvignon and Kyoho (OIV, 2017), and it is one of the most important red grape varieties grown in Spain. This cultivar is characterized by subtle aroma, producing wines with fruity and spicy flavors, low acidity, and low tannins. However, wine organoleptic characteristics are highly determined by the characteristics of the grapes used for its production. Therefore, changes in grapevine growing conditions that affect berry composition are also likely to impact the wine produced. Grape quality is a complex trait that mainly refers to berry composition, including sugars, organic acids (malic and tartaric acid), amino acids, and a wide range of secondary metabolites such as phenolic compounds, aromas, and aroma precursors (Conde et al., 2007). Among the factors that affect berry content at harvest, climate parameters, and notably temperature, play a prominent role.

The Intergovernmental Panel on Climate Change (IPCC) has pointed out the ineluctable increase in the temperature worldwide and has identified climate change as an important threat to global food supply. Indeed, greenhouse gas (GHG) emissions have increased greatly during the last decades, affecting the equilibrium of biogeochemical cycles and, hence, the composition of the atmosphere (IPCC, 2013). Some of the consequences of the rise in the atmospheric concentration of GHG are at global climate level, as high levels of GHG block heat loss of the planet, thus contributing to the so-called “greenhouse effect” and to global warming. Another effect of the anthropogenic GHG emissions is the increase in the concentration of CO_2_ in the atmosphere (IPCC, 2014). The IPCC has carried out different reports from scientific knowledges on climate change to determine the magnitude of these changes according to different scenarios. Some of the predictions for global mean temperature in 2100 show an increment between 2.2 ± 0.5 and 3.7 ± 0.7°C, and the estimations for future atmospheric CO_2_ concentration are between 669.7 and 935.9 ppm (scenarios RCP 6.0 and RCP 8.5 of the IPCC) (IPCC, 2013).

Research on the response of grapevine to the abovementioned environmental factors has concluded that high temperature affects the phenology of grapevine, as well as grape berry development and ripening, hastening the latter (Jones and Davis, 2000; Duchêne and Schneider, 2005; Webb et al., 2007; Keller, 2010; Martínez-Lüscher et al., 2016a) and affecting both primary and secondary metabolisms. Berry sugar accumulation is altered under climate change conditions (Duchêne and Schneider, 2005; Kuhn et al., 2014), while malic acid degradation is enhanced by high temperatures (Orduña, 2010; Carbonell-Bejerano et al., 2013), and by its combination with elevated CO_2_ (Salazar Parra et al., 2010; Martínez-Lüscher et al., 2016b). Secondary metabolism is also sensitive to high temperatures, particularly the flavonoid and aroma precursor pathways, as evidenced by transcriptomic, proteomic, and metabolomic approaches (Spayd et al., 2002; Tarara et al., 2008; Azuma et al., 2012; Rienth et al., 2014; Martínez-Lüscher et al., 2016a; Lecourieux et al., 2017, 2020; Arrizabalaga et al., 2018), thus affecting the balance of berry quality-related compounds at ripeness (Sadras and Moran, 2012; Kuhn et al., 2014). Whereas increased temperature has been consistently shown to reduce anthocyanin levels (Spayd et al., 2002; Mori et al., 2007; Tarara et al., 2008; Azuma et al., 2012), the effect of CO_2_ on these compounds is more controversial, with some authors reporting no effect (Gonçalves et al., 2009), while others reported an increase in anthocyanin levels (Kizildeniz et al., 2015). Finally, the decoupling in the accumulation of anthocyanins and sugars, thus leading to an imbalance between these two compounds at maturity, has been also described as a consequence of elevated temperature (Sadras and Moran, 2012; Kuhn et al., 2014).

The studies on the impact of multiple stress factors associated with climate change on grape development and composition remain limited due to their complexity. However, in the future, plants will be exposed to multiple elements of environmental change simultaneously, their combined effects being not always additive. Indeed, the impact of an increase in atmospheric CO_2_ concentration on plant carbon metabolism, notably on photosynthesis and respiration processes, has been previously reported (Dusenge et al., 2019). Zhao et al. (2019) also demonstrated that photosynthesis of *in vitro* grape plantlet was promoted by elevated CO_2_ concentration. These modifications may directly affect primary and secondary plant metabolism, thus leading to substantial changes in fruit composition. However, Dusenge et al. (2019) showed that some effects of increased CO_2_ concentration on carbon plant metabolism could be minimized by elevated temperatures and highlighted the interest to study these climatic parameters together, both separately and in combination.

Regarding grapevine, previous studies on the effect of elevated CO_2_ and elevated temperature indicated that the combination of these two factors can affect plant phenology, thus hastening grape ripening (Salazar-Parra, 2011; Martínez-Lüscher et al., 2016b) and decreasing malic acid concentration (Leibar et al., 2016). Nevertheless, the authors reported also a decrease in grape total anthocyanin concentration at maturity. Therefore, in order to avoid detrimental wine traits, it is important to determine how grape characteristics will be affected by the abovementioned climate change-related factors, acting not only individually but also combined, and to investigate approaches to mitigate the undesired effects, ensuring the sustainability of this crop. Among the strategies proposed to adapt viticulture to a changing environment, the genetic improvement and adaptation of elite and autochthonous varieties are very relevant to keep their intrinsic varietal values and typicity (Cunha et al., 2009; Keller, 2010; Carbonell-Bejerano et al., 2019). Accordingly, the selection of grapevine varieties and clones within the same variety with longer ripening periods has been suggested as a valuable tool to exploit in order to find accessions keeping current traits under future climate conditions (Duchêne et al., 2010).

Certain varieties used for wine production are tightly linked to specific production areas. This is the case of Tempranillo in Spain, Merlot in France, Sangiovese in Italy, or Fernão Pires in Portugal (Sefc et al., 2003). Tempranillo requires warm, sunny days to reach full maturity but also cool nights to keep its natural acidity. Maturity occurs fairly early in comparison with Grenache, the variety commonly blended with Tempranillo. Nevertheless, the constant increase in temperature and CO_2_ levels in the future could significantly shift the optimal maturation conditions in these areas, which would have a significant effect on berry quality. For these reasons, successfully exploiting the intravarietal diversity has the potential to face with the putative negative impacts of climate change, as it would allow to keep the culture of traditional varieties. The research done so far in Tempranillo includes the identification and characterization of a large number of clones (49 certified clones), which differ either in reproductive or vegetative traits (Ibáñez et al., 2015), making possible the research of clones better adapted to future climate conditions.

In previous studies, we have reported that Tempranillo clones differ in their response to elevated temperature in terms of sugar and anthocyanin accumulation (Arrizabalaga et al., 2018). We have also demonstrated that Tempranillo clones presented differences in their phenological development, notably in terms of vegetative production and carbon partitioning into organs, in response to elevated CO_2_ and increased temperature (Arrizabalaga-Arriazu et al., 2020). However, plants showed signs of photosynthetic acclimation to CO_2_, especially when they were exposed to elevated CO_2_ combined with high temperature conditions, joining the idea that effects of increasing CO_2_ concentration on plant carbon metabolism may be modulated by elevated temperatures as mentioned above. In this context, the objective of this work was to study the effects of increased temperature and rise in atmospheric CO_2_, alone or in combination, on berry development and composition of five different clones of *Vitis vinifera* cv. Tempranillo exhibiting different lengths of their reproductive cycle. The study was focused on the evolution of must composition (malic acid, sugars, and amino acids) and skin total anthocyanins throughout the ripening period, aiming to assess whether the impact of temperature and CO_2_ differs among different clones of Tempranillo. We hypothesized that clones within the same variety that show differences in phenological development may respond in a different manner to the abovementioned climate change-related factors, in terms of grape composition. Indeed, grape composition could be less affected by future climatic conditions in clones with longer reproductive cycle than in early ripening clones, since in the former the ripening would take place under cooler conditions. Few studies have assessed the performance of different clones of the same cultivar to climate conditions foreseen by the end of this century. The novelty of this work is the assessment of three-way interactions among Tempranillo clones, elevated temperature, and high atmospheric CO_2_. The information obtained can be useful to evaluate whether genetic diversity can be an appropriate tool to design adaption strategies for viticulture in the future.

## Materials and Methods

### Plant Material: Origin and Development

Dormant cuttings of five clones of grapevine (*V. vinifera* L.) cv. Tempranillo were obtained from the germplasm bank of three institutions: RJ43, CL306 (both clones widely cultivated in Spain), and T3 were obtained from Estación de Viticultura y Enología de Navarra (EVENA), located in Olite, Navarra (Spain); 1084 was obtained from the Institute of Sciences of Vine and Wine, located in “La Grajera,” La Rioja (Spain); and VN31 was facilitated by Vitis Navarra, located in Larraga, Navarra (Spain). The reproductive cycle length of these clones had been characterized previously, presenting differences among them: VN31 and 1084 have been described as long reproductive cycle accessions (Arrizabalaga et al., 2018; Vitis Navarra, 2019), CL306 and T3 have been defined as short cycle accessions (Rubio and Yuste, 2005; Vicente Castro, 2012; Cibriáin et al., 2018), and RJ43 is considered to have an intermediate reproductive cycle length (EVENA, 2009).

Fruit-bearing cuttings were obtained as described in Arrizabalaga et al. (2018) with minor modifications. They were manipulated to develop a single berry bunch and they were grown at the same conditions as described in Arrizabalaga et al. (2018) from March to May 2016, when the fruit set took place. Then, plants were transferred to 13 L pots with 2:1 peat:perlite mixture (v/v) and moved afterward to temperature gradient greenhouses (TGGs) located at the campus of the University of Navarra (42°48′N, 1°40′W; Pamplona, Navarra, Spain). The irrigation, both before fruit set and in the TGGs, was carried out using the nutritive solution described by Ollat et al. (1998).

### Treatments and Plant Growth in Temperature Gradient Greenhouses

Treatment application was conducted in TGGs, a research-oriented structure for plant growth with semicontrolled conditions, taking into consideration natural environmental conditions. Each TGG is divided into three temperature modules which create a gradient of temperature (from module 1 of ambient temperature to module 3 of ambient temperature + 4°C), as the air heats up when passing through them (Morales et al., 2014). The temperature records during the experiment are included in Supplementary Figure 1. In addition, CO_2_ can be injected inside the TGGs, modifying the air CO_2_ concentration.

An equal number of plants of each clone were placed in the first and the third module of four TGGs, leaving the central module free of plants. Half of the plants (those located in modules 1) grew at ambient temperature (*T*), corresponding to the ambient temperature outdoors, while the other half of the plants (those located in modules 3) grew under ambient plus 4°C warmer temperature (*T* + 4). Besides, the air CO_2_ concentration was modified in two out of the four TGGs, resulting in half of the plants growing at current atmospheric CO_2_ concentration (ca. 400 ppm; ACO_2_) and the other half under increased air CO_2_ concentration (700 ppm; ECO_2_). Therefore, plants grew under four different CO_2_ and temperature conditions: (i) ambient temperature and ambient CO_2_ (*T*/ACO_2_), (ii) ambient temperature and elevated CO_2_ (*T*/ECO_2_), (iii) elevated temperature and ambient CO_2_ (*T* + 4/ACO_2_), and (iv) elevated temperature and elevated CO_2_ (*T* + 4/ECO_2_). The treatments were applied from fruit set (2016, May) to berry maturity (2016, September), which was considered to occur when the total soluble solid (TSS) content of the berries was ca. 22°Brix, each plant being measured individually (°Brix was determined on grape juice, after pressing entire grape berries, using a hand refractometer with temperature compensation). The number of plants per treatment and clone was 8.

### Phenological Development and Berry Size

The length of the phenological development of grapes was determined for each plant by individually recording the dates corresponding to fruit set, mid-veraison (half of the berries in the bunch had started to change color), and maturity. The time intervals between fruit set and mid-veraison and between mid-veraison and maturity were calculated. Fruit set and mid-veraison were determined visually, and maturity was considered to be reached when the levels of TSS of two berries of each bunch were, at least, 22°Brix. This analysis was done periodically for each bunch every 2–3 days during the last weeks of development of the berries.

In order to carry out the different analyses of berries, five sampling points were determined: (i) onset of veraison, when berries had already softened and just began to color; (ii) mid-veraison (determined as described previously in this section), at this stage, berries with the same proportion of colored skin surface were sampled (ca. 50%); (iii) 1 week after mid-veraison; (iv) 2 weeks after mid-veraison; and (v) maturity, determined as described previously in this section. At the onset of veraison, three to four berries were sampled from each bunch, three berries at mid-veraison, 1 week after mid-veraison and 2 weeks after mid-veraison, and 10 berries at maturity. Each plant was sampled individually when reaching the desired stage. In order to avoid differences in berry composition due to their position in the bunch, all the berries were taken from the top and middle portion of the bunch, which allocate the highest number of berries. The diameter was measured with a caliper and berries were frozen in liquid nitrogen and stored at −80°C until analyses.

### Berry Analyses Preparation

Analyses were carried out by doing pools of berries (3–10 berries per bunch, see above, from two or three different plants per pool, four pools per treatment and clone). Berries were manually peeled and separated into skin, pulp, and seeds. Fresh skins, pulps, and seeds were weighed and the data obtained were used to determine the relative skin mass (relation between skin fresh weight and total berry fresh weight, expressed as a percentage). The pulp was crushed to obtain the must, which was centrifuged and used for sugar, malic acid, and amino acid analyses. The skin was freeze-dried in an Alph1-4, lyophilizer (CHRIST, Osterode, Germany), weighed to calculate the water content, and ground into powder using an MM200 ball grinder (Retsch, Haan, Germany) for carrying out the anthocyanin analyses.

### Total Soluble Solids, Sugar, Malic Acid, and Amino Acid Profile Analyses

Total soluble solids content in the must was measured using a Handheld Digital Refractometer (RD110). Sugar (glucose and fructose) concentration was determined enzymatically using an automated absorbance microplate reader (Elx800UV, BioTek Instruments, Inc., Winooski, VT, United States) using the Glucose/Fructose kit from BIOSENTEC (Toulouse, France) according to the manufacturer. Malic acid was determined with a Bran and Luebbe TRAACS 800 autoanalyzer (Bran & Luebbe, Plaisir, France) as described in detail in Bobeica et al. (2015).

For the amino acid analysis, samples were derivatized with the AccQFluor Reagent (6-aminoquinolyl-*N*-hydroxy-succinimidyl-carbamate, Waters, Milford, MA, United States) (Cohen and Michaud, 1993) as described by Hilbert et al. (2015). The products of this reaction were analyzed with an UltiMate 3000 UHPLC system (Thermo Electron SAS, Waltham, MA, United States) equipped with an FLD-3000 Fluorescence Detector (Thermo Electron SAS, Waltham, MA, United States). Amino acids were separated using as eluents sodium acetate buffer (eluent A, 140 mM at pH 5.7), acetonitrile (eluent B), and water (eluent C) at 37°C and 0.5 ml min^–1^ through an AccQTag Ultra column, 2.1 mm × 100 mm, 1.7 μm (Waters, Milford, MA, United States) according to Habran et al. (2016). The concentration and identification of each compound was determined through a chromatographic analysis as described in Pereira et al. (2006), using an excitation wavelength of 250 nm and an emission wavelength of 395 nm. Samples were loaded alternated with control samples as in Torres et al. (2017).

### Total Skin Anthocyanin Analyses

Anthocyanin analyses were carried out according to Torres et al. (2017) and described in detail by Acevedo de la Cruz et al. (2012) and Hilbert et al. (2015) with minor changes. In brief, ground dried skins were treated with methanol containing 0.1% HCl (v/v), in order to extract the pigments, and filtered using a polypropylene syringe filter of 0.45 μm (Pall Gelman Corp., Ann Arbor, MI, United States). The obtained extracts were separated using a Syncronis C18, 2.1 mm × 100 mm, 1.7 μm column (Thermo Fisher Scientific, Waltham, MA, United States) and analyzed with an UltiMate 3000 UHPLC system (Thermo Electron SAS, Waltham, MA, United States) equipped with DAD-3000 diode array detector (Thermo Electron SAS, Waltham, MA, United States). The wavelength used for recording the chromatographic profiles was 520 nm and the standard was malvidin-3-*O*-glucoside (Extrasynthese, Genay, France). The peak areas of chromatograms were calculated using the Chromeleon software (version 7.1) (Thermo Electron SAS, Waltham, MA, United States). The concentration of total anthocyanins was calculated as the sum of the concentration of individual anthocyanins. In order to evaluate the impact of environmental factors on the balance between anthocyanins and sugars, the ratio between the concentration of anthocyanins and the level of TSS was calculated at maturity.

### Statistical Analysis

The data were statistically analyzed using R (3.5.1), carrying out a three-way ANOVA (clone, temperature, and CO_2_ concentration) and a Fisher’s least significant difference (LSD) was carried out as a *post hoc* test when statistically significant differences were found (*P* < 0.05).

## Results

### Phenological Development

**FIGURE 1:**
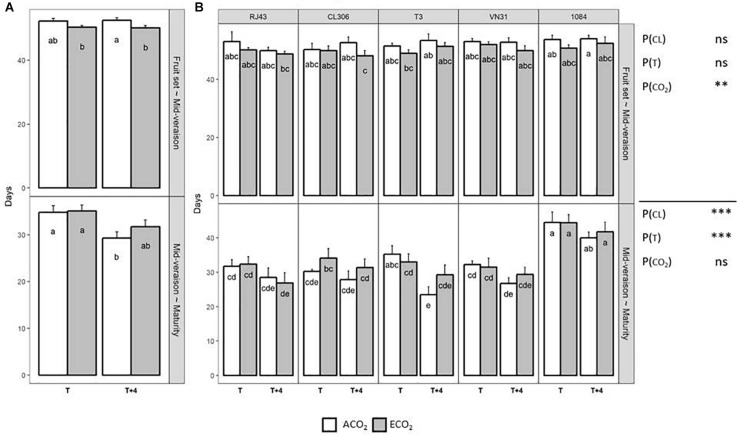
Elapsed time between fruit set and mid-veraison and between mid-veraison and maturity of the five Tempranillo clones grown under four temperature/CO_2_ regimes: ambient temperature (*T*) or ambient temperature + 4°C (*T* + 4), combined with ambient CO_2_ (ca. 400 ppm, ACO_2_) or elevated CO_2_ (700 ppm, ECO_2_). Results (values are means ± SE) are presented according to the temperature (*T* or *T* + 4) and CO_2_ regime (ACO_2_ or ECO_2_), **(A)** considering all the clones as altogether (*n* = 20–40) and **(B)** considering each clone individually (*n* = 4–8). Means with letters in common within each chart **(A,B)** and period are not significantly different according to the least significant difference (LSD) test (*P* > 0.05). Probability values (*P*) for the main effects of clone, *P*_(CL)_; temperature, *P*_(_*_*T*_*_)_; and CO_2_, *P*_(CO__2__)_. ^∗∗∗^*P* < 0.001; ^∗∗^*P* < 0.01; ns, not significant. All probability values for the interactions of factors [*P*_(CL ×_ *_*T*_*_)_, *P*_(CL × *CO*__2__)_, *P*_(_*_*T*_*_× *CO*__2__)_, and *P*_(CL ×_ *_*T*_*_× *CO*__2__)_] were statistically not significant (*P* > 0.05).

In general, elevated CO_2_ had a higher impact on grape phenology in the period comprised between fruit set to mid-veraison, whereas ripening (mid-veraison to maturity) was more affected by elevated temperature (Figure 1A). The number of days elapsed between fruit set and mid-veraison was slightly, but significantly, shortened by ECO_2_, and especially when it was combined with *T* + 4 (Figure 1A). However, this hastening effect of CO_2_ was nullified between mid-veraison and maturity, whereas the increase of temperature reduced significantly this period in up to 5 days. Also, the duration from mid-veraison to maturity was affected significantly by the clone identity, mainly because 1084 needed more time to reach maturity, regardless of the growing condition (Figure 1B). Although significant interactions among factors were not detected, it is worth pointing out the significant difference in the elapsed days between mid-veraison and maturity of T3 plants grown under *T* + 4/ACO_2_ compared with *T*/ACO_2_, as maturity was reached 12 days earlier when *T* + 4 was applied.

### Berry Characteristics

Berry diameter differed significantly among clones at the onset of veraison, mid-veraison, and maturity (Table 1). It was also modified by temperature, decreasing under *T* + 4 2 weeks after mid-veraison and at maturity. By contrast, the CO_2_ level did not markedly affect this berry characteristic. The only noteworthy interaction among the parameters was at mid-veraison, when the effect of ECO_2_ was different depending on the temperature regime and the clone (triple interaction).

**TABLE 1:** Grape berry diameter of the five Tempranillo clones (RJ43, CL306, T3, VN31, and 1084) grown under four temperature/CO_2_ regimes: ambient temperature (T) or ambient temperature + 4°C (*T* + 4), combined with ambient CO_2_ (ca. 400 ppm, ACO_2_) or elevated CO_2_ (700 ppm, ECO_2_).

Berry diameter (mm)							
			Onset of veraison	Mid-veraison	1 week after mid-veraison	2 weeks after mid-veraison	Maturity
RJ43			10.51 ± 0.18ab	10.78 ± 0.23bc	11.36 ± 0.21b	13.19 ± 1.09a	12.71 ± 0.17ab
CL306			10.11 ± 0.16b	10.57 ± 0.13c	11.36 ± 0.19b	12.07 ± 0.19a	12.33 ± 0.20b
T3			10.86 ± 0.20a	11.11 ± 0.19b	11.63 ± 0.23ab	12.40 ± 0.22a	13.11 ± 0.17a
VN31			10.77 ± 0.15a	11.19 ± 0.13ab	13.00 ± 1.25a	13.81 ± 1.07a	13.07 ± 0.16a
1084			10.86 ± 0.23a	11.60 ± 0.19a	12.10 ± 0.20ab	12.16 ± 0.30a	12.88 ± 0.25a
*T*			10.75 ± 0.12a	11.18 ± 0.11a	11.85 ± 0.13a	13.39 ± 0.60a	13.14 ± 0.13a
*T* + 4			10.49 ± 0.13a	10.92 ± 0.13a	11.93 ± 0.52a	12.07 ± 0.15b	12.50 ± 0.10b
ACO_2_			10.60 ± 0.10a	11.07 ± 0.12a	12.16 ± 0.51a	12.42 ± 0.11a	12.68 ± 0.13a
ECO_2_			10.64 ± 0.14a	11.03 ± 0.13a	11.62 ± 0.15a	13.03 ± 0.63a	12.96 ± 0.13a
RJ43	*T*	ACO_2_	10.93 ± 0.49abc	11.18 ± 0.47abcd	11.55 ± 0.24b	12.70 ± 0.15bc	13.01 ± 0.52bcde
		ECO_2_	10.39 ± 0.36bcd	10.44 ± 0.43def	11.45 ± 0.53b	16.43 ± 4.35ab	12.82 ± 0.13cdef
	*T* + 4	ACO_2_	10.50 ± 0.24bc	10.18 ± 0.21ef	10.73 ± 0.44b	11.63 ± 0.18c	12.56 ± 0.42defg
		ECO_2_	10.21 ± 0.33cd	11.32 ± 0.53abcd	11.70 ± 0.37b	12.01 ± 0.11c	12.46 ± 0.24defg
CL306	T	ACO_2_	10.03 ± 0.18cd	10.52 ± 0.20def	11.35 ± 0.17b	12.11 ± 0.25c	11.84 ± 0.56g
		ECO_2_	10.32 ± 0.34cd	11.04 ± 0.07abcde	11.93 ± 0.22b	12.31 ± 0.37c	12.95 ± 0.27bcdef
	*T* + 4	ACO_2_	10.68 ± 0.18abc	10.71 ± 0.26cdef	11.44 ± 0.54b	12.69 ± 0.16bc	12.52 ± 0.34defg
		ECO_2_	9.42 ± 0.20d	10.02 ± 0.22f	10.71 ± 0.31b	11.18 ± 0.26c	12.03 ± 0.19fg
T3	*T*	ACO_2_	10.72 ± 0.33abc	11.16 ± 0.04abcd	12.09 ± 0.09b	12.45 ± 0.19bc	13.22 ± 0.14abcd
		ECO_2_	11.59 ± 0.24a	11.87 ± 0.09a	12.46 ± 0.28b	13.30 ± 0.23abc	13.97 ± 0.23a
	*T* + 4	ACO_2_	10.29 ± 0.34cd	10.74 ± 0.56cdef	11.17 ± 0.47b	11.95 ± 0.58c	12.43 ± 0.14defg
		ECO_2_	10.83 ± 0.45abc	10.68 ± 0.35cdef	10.82 ± 0.44b	11.91 ± 0.34c	12.81 ± 0.20cdef
VN31	*T*	ACO_2_	10.94 ± 0.31abc	11.25 ± 0.19abcd	11.97 ± 0.57b	12.67 ± 0.08bc	13.57 ± 0.13abc
		ECO_2_	10.90 ± 0.35abc	11.57 ± 0.23abc	11.77 ± 0.75b	16.79 ± 4.33a	13.21 ± 0.25abcd
	*T* + 4	ACO_2_	10.68 ± 0.29abc	11.16 ± 0.30abcd	16.81 ± 4.86b	13.04 ± 0.42abc	12.54 ± 0.25defg
		ECO_2_	10.55 ± 0.32bc	10.79 ± 0.21cdef	11.47 ± 0.39b	12.76 ± 0.29bc	12.97 ± 0.44bcdef
1084	*T*	ACO_2_	10.87 ± 0.24abc	11.93 ± 0.33a	12.32 ± 0.54b	12.63 ± 0.60bc	13.02 ± 0.38bcde
		ECO_2_	10.80 ± 0.53abc	10.87 ± 0.41bcdef	11.58 ± 0.50b	12.47 ± 0.22bc	13.84 ± 0.45ab
	*T* + 4	ACO_2_	10.38 ± 0.52bcd	11.86 ± 0.18a	12.15 ± 0.35a	12.36 ± 0.15c	12.12 ± 0.33efg
		ECO_2_	11.38 ± 0.54ab	11.76 ± 0.41ab	12.35 ± 0.18b	11.19 ± 0.95c	12.57 ± 0.52defg
*P*_(CL)_			*	***	ns	ns	*
*P*_(_*_*T*_*_)_			ns	ns	ns	*	***
*P*(_*CO*__2_)			ns	ns	ns	ns	ns
*P*_(CL ×_ *_*T*_*_)_			ns	ns	ns	ns	ns
*P*_(CL × *CO*__2__)_			ns	ns	ns	ns	ns
*P*_(_*_*T*_*_× *CO*__2__)_			ns	ns	ns	ns	ns
*P*_(CL ×_ *_*T*_*_× *CO*__2__)_			ns	**	ns	ns	ns

Results (values are means ± SE) are shown according to clone identity (*n* = 16), temperature regime (*n* = 40), CO_2_ concentration (*n* = 40), and the combined factors (*n* = 4). Means with letters in common within the same stage and factor (clone, temperature, CO_2_, or their interactions) are not significantly different according to the least significant difference (LSD) test (*P* > 0.05). Probability values (*P*) for the main effects of clone, *P*_(CL)_; temperature, *P*_(_*_*T*_*_)_; CO_2_, *P*_(CO__2__)_; and their interactions, *P*_(CL ×_*_*T*_*_)_, *P*_(CL × CO__2__)_, *P*_(_*_*T*_*_× CO__2__)_, and *P*_(CL ×_*_*T*_*_× CO__2__)_. ***P < 0.001; **P < 0.01; *P < 0.05; ns, not significant.

In general, berries from all the studied clones presented similar relative skin mass throughout the experiment except at maturity, when 1084 had significantly lower values than CL306, T3, and VN31 (Table 2). Relative skin masses were higher 1 and 2 weeks after mid-veraison in grapes developed under *T* + 4 compared with those grown at *T*. Grapes under ECO_2_ had a lower relative skin mass than ACO_2_ at the onset of veraison, but higher 1 and 2 weeks after mid-veraison. At maturity, the T3 clone was the most affected one by the increase in temperature of *T* + 4, especially when combined with ACO_2_.

**TABLE 2:** Relative skin mass (%) in grape berries of the five Tempranillo clones (RJ43, CL306, T3, VN31, and 1084) grown under four temperature/CO_2_ regimes: ambient temperature (*T*) or ambient temperature + 4°C (*T* + 4), combined with ambient CO_2_ (ca. 400 ppm, ACO_2_) or elevated CO_2_ (700 ppm, ECO_2_).

Relative skin mass (%)							
			Onset of veraison	Mid-veraison	1 week after mid-veraison	2 weeks after mid-veraison	Maturity
RJ43			16.76 ± 1.30a	15.60 ± 0.89a	14.02 ± 0.57a	15.02 ± 0.76a	16.50 ± 0.68ab
CL306			15.24 ± 0.66a	14.27 ± 0.40ab	14.73 ± 0.88a	17.02 ± 1.57a	17.51 ± 0.76a
T3			14.68 ± 0.64a	13.76 ± 0.70ab	14.20 ± 0.63a	14.83 ± 1.03a	16.88 ± 0.84a
VN31			15.43 ± 0.53a	13.90 ± 0.75ab	14.79 ± 0.55a	15.62 ± 0.90a	17.72 ± 0.87a
1084			14.57 ± 0.58a	13.24 ± 0.44b	14.35 ± 0.60a	14.81 ± 1.61a	14.41 ± 0.78b
*T*			15.48 ± 0.40a	14.36 ± 0.37a	13.85 ± 0.31b	13.72 ± 0.32b	16.03 ± 0.52a
*T* + 4			15.19 ± 0.59a	13.94 ± 0.48a	14.98 ± 0.47a	17.31 ± 1.01a	17.22 ± 0.51a
ACO_2_			16.56 ± 0.57a	13.74 ± 0.46a	13.67 ± 0.42b	14.35 ± 0.77b	16.81 ± 0.55a
ECO_2_			14.11 ± 0.33b	14.57 ± 0.39a	15.17 ± 0.37a	16.56 ± 0.75a	16.48 ± 0.5a
RJ43	*T*	ACO_2_	17.79 ± 0.71ab	15.33 ± 0.91abc	12.24 ± 0.35c	12.33 ± 0.57c	15.80 ± 1.19bcdef
		ECO_2_	13.75 ± 1.16bcd	15.51 ± 2.80abc	14.52 ± 0.79abc	14.63 ± 0.86bc	16.04 ± 1.72abcdef
	*T* + 4	ACO_2_	20.68 ± 4.63a	16.15 ± 2.06a	12.66 ± 0.18bc	15.87 ± 1.39bc	17.12 ± 1.93abcdef
		ECO_2_	14.81 ± 0.89bcd	15.41 ± 1.64abc	16.66 ± 1.27a	17.26 ± 2.08abc	17.02 ± 0.80abcdef
CL306	*T*	ACO_2_	16.64 ± 1.35abc	12.91 ± 0.41abc	13.90 ± 0.29abc	14.07 ± 1.08bc	18.16 ± 2.76abcd
		ECO_2_	14.48 ± 0.44bcd	15.75 ± 0.86ab	12.55 ± 0.74bc	15.70 ± 0.98bc	17.84 ± 1.15abcd
	*T* + 4	ACO_2_	15.04 ± 1.59bcd	13.94 ± 0.74abc	16.64 ± 3.13a	15.30 ± 1.59bc	18.60 ± 1.64abc
		ECO_2_	14.79 ± 1.80bcd	14.46 ± 0.63abc	15.84 ± 1.35ab	22.58 ± 5.00a	15.60 ± 0.71bcdef
T3	*T*	ACO_2_	17.56 ± 1.15abc	15.18 ± 1.05abc	14.48 ± 1.75abc	13.10 ± 0.82bc	14.21 ± 1.50def
		ECO_2_	12.05 ± 0.81d	13.18 ± 0.74abc	13.26 ± 0.65abc	13.29 ± 1.53bc	15.10 ± 0.29cdef
	*T* + 4	ACO_2_	15.17 ± 0.80bcd	12.13 ± 2.36bc	12.47 ± 0.60bc	15.82 ± 3.01bc	20.05 ± 0.91a
		ECO_2_	13.94 ± 0.52bcd	14.54 ± 0.83abc	16.58 ± 0.90a	17.88 ± 2.06abc	18.16 ± 1.95abcd
VN31	*T*	ACO_2_	17.32 ± 1.59abc	14.92 ± 1.19abc	14.36 ± 0.85abc	13.66 ± 0.93bc	15.99 ± 1.79abcdef
		ECO_2_	14.85 ± 0.75bcd	13.44 ± 0.50abc	14.78 ± 0.53abc	15.82 ± 0.84bc	19.61 ± 1.34ab
	*T* + 4	ACO_2_	15.43 ± 0.41bcd	11.81 ± 2.22c	13.36 ± 1.07abc	14.09 ± 2.50bc	17.34 ± 2.17abcde
		ECO_2_	14.10 ± 0.65bcd	15.44 ± 1.42abc	16.65 ± 1.42a	19.57 ± 0.95ab	17.94 ± 1.80abcd
1084	*T*	ACO_2_	15.59 ± 0.67bcd	13.07 ± 0.38abc	12.93 ± 1.14bc	12.34 ± 0.35c	14.10 ± 1.16def
		ECO_2_	14.78 ± 1.10bcd	14.35 ± 1.08abc	15.50 ± 1.45abc	12.75 ± 0.50bc	12.03 ± 1.08f
	*T* + 4	ACO_2_	14.38 ± 0.92bcd	11.97 ± 0.91bc	13.66 ± 0.95abc	17.12 ± 6.61abc	17.07 ± 1.73abcdef
		ECO_2_	13.55 ± 1.85cd	13.57 ± 0.82abc	15.31 ± 1.14abc	17.02 ± 0.96abc	13.24 ± 0.94ef
*P*_(CL)_			ns	ns	ns	ns	*
*P*_(_*_*T*_*_)_			ns	ns	*	**	ns
*P*_(CO__2__)_			***	ns	**	ns	ns

Results (values are means ± SE) are shown according to the clone (*n* = 16), temperature (*n* = 40), CO_2_ concentration (*n* = 40), and the combined factors (*n* = 4). Means with letters in common within the same stage and factor (clone, temperature, CO_2_, or their interactions) are not significantly different according to the LSD test (*P* > 0.05). Probability values (*P*) for the main effects of clone, *P*_(CL)_; temperature, *P*_(_*_*T*_*_)_; and CO_2_, *P*_(CO__2__)_. ***P < 0.001; **P < 0.01; *P < 0.05; ns, not significant. All probability values for the interactions of factors [*P*_(CL ×_*_*T*_*_)_, *P*_(CL × CO__2__)_, *P*_(_*_*T*_*_× CO__2__)_, and *P*_(CL ×_*_*T*_*_× CO__2__)_] were statistically not significant (*P* > 0.05).

### Malic Acid

**FIGURE 2:**
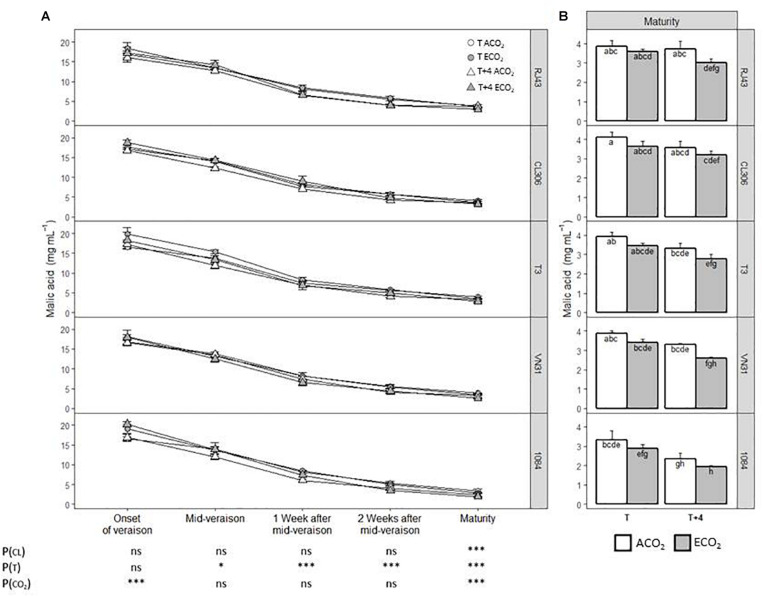
Malic acid concentration of the five Tempranillo clones grown under four temperature/CO_2_ regimes: ambient temperature (*T*) or ambient temperature + 4°C (*T* + 4), combined with ambient CO_2_ (ca. 400 ppm, ACO_2_) or elevated CO_2_ (700 ppm, ECO_2_). Data (values are means ± SE, *n* = 4) are presented according to temperature (*T* or *T* + 4) and CO_2_ regime (ACO_2_ or ECO_2_) **(A)** throughout ripening and **(B)** at maturity. Data are presented according to the temperature (*T* or *T* + 4) and CO_2_ regime (ACO_2_ or ECO_2_) and considering each clone individually. Means with letters in common are not significantly different according to the LSD test (*P* > 0.05). Probability values (*P*) for the main effects of clone, *P*_(CL)_; temperature, *P*_(_*_*T*_*_)_; and CO_2_, *P*_(CO__2__)_. ***P < 0.001; *P < 0.05; ns, not significant. All probability values for the interactions of factors [*P*_(CL ×_ *_*T*_*_)_, *P*_(CL × *CO*__2__)_, *P*_(_*_*T*_*_× *CO*__2__)_, and *P*_(CL ×_ *_*T*_*_× *CO*__2__)_] were statistically not significant (*P* > 0.05).

The evolution of malic acid concentration throughout the ripening process was not affected by the clone identity, decreasing in a similar manner in all of them until maturity (Figure 2A). However, at maturity, the 1084 accession had the lowest malic acid levels and CL306 the highest (Figure 2B). Considering all the clones as a whole, *T* + 4 decreased significantly malic acid from mid-veraison onward with respect to *T*, while ECO_2_ raised acid malic significantly at the onset of veraison and reduced it at maturity compared with ACO_2_ (Figure 2A). For all the clones studied, grapes developed under current situation (*T*/ACO_2_) presented significantly higher levels of malic acid at maturity than those developed under climate change conditions (*T* + 4/ECO_2_) (Figure 2B). In the case of 1084, this difference was observed with plants grown at *T* + 4, regardless of the CO_2_ regime. Globally, there were no significant interactions among factors.

### Sugars and Total Soluble Solids

**FIGURE 3:**
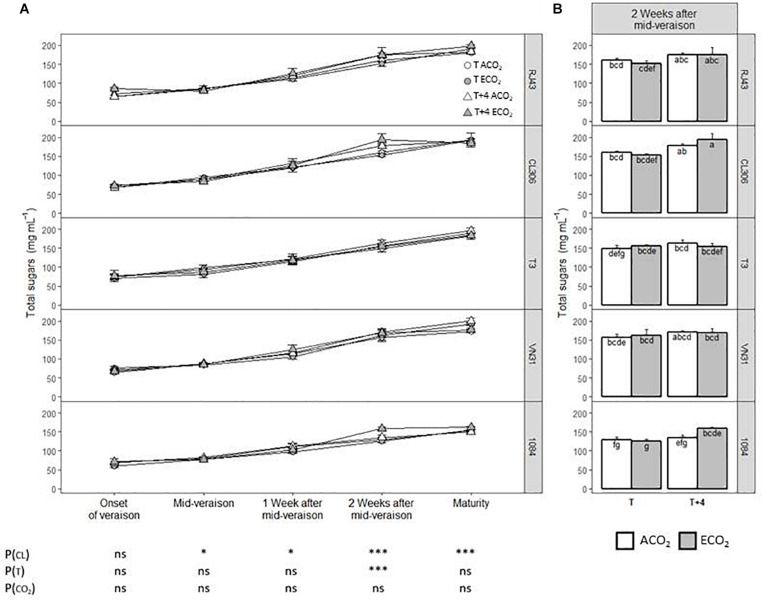
Sugar concentration in berries of the five Tempranillo clones grown under four temperature/CO_2_ regimes: ambient temperature (*T*) or ambient temperature + 4°C (*T* + 4), combined with ambient CO_2_ (ca. 400 ppm, ACO_2_) or elevated CO_2_ (700 ppm, ECO_2_). Data are presented according to temperature (*T* or *T* + 4) and CO_2_ regime (ACO_2_ or ECO_2_) and considering each clone individually (values are means ± SE, *n* = 4): **(A)** throughout ripening and **(B)** 2 weeks after mid-veraison. Means with letters in common are not significantly different according to LSD test (*P* > 0.05). Probability values (*P*) for the main effects of clone, *P*_(CL)_; temperature, *P*_(_*_*T*_*_)_; and CO_2_, *P*_(CO__2__)_. ***P < 0.001; *P < 0.05; ns, not significant. All probability values for the interactions of factors [*P*_(CL ×_ *_*T*_*_)_, *P*_(CL × *CO*__2__)_, *P*_(_*_*T*_*_× *CO*__2__)_, and *P*_(CL ×_ *_*T*_*_× *CO*__2__)_] were statistically not significant (*P* > 0.05).

In general, the level of sugars (glucose and fructose) depended significantly on the clone from mid-veraison onward, being strongly affected by this factor 2 weeks after mid-veraison and at maturity (Figure 3A). Notably, 2 weeks after mid-veraison, the most contrasted clones were 1084 and CL306, with the lowest and highest sugar levels, respectively (Figure 3B). The *T* + 4 treatment increased significantly the sugar concentration 2 weeks after mid-veraison compared with *T*, whereas the atmospheric CO_2_ level did not have any effect on this parameter. Nonetheless, there were no significant interactions among the factors considered. The trends observed for total soluble solids at maturity (Supplementary Figure 2) were quite similar to those observed for the sum of glucose and fructose at this stage (Figure 3), clone 1084 being the one with the lowest TSS levels.

### Amino Acids

**FIGURE 4:**
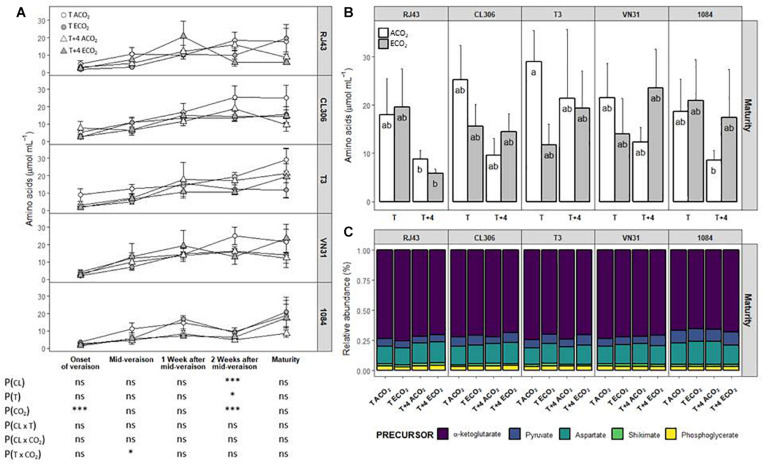
Amino acid concentration in berries of the five Tempranillo clones grown under four temperature/CO_2_ regimes: ambient temperature (*T*) or ambient temperature + 4°C (*T* + 4), combined with ambient CO_2_ (ca. 400 ppm, ACO_2_) or elevated CO_2_ (700 ppm, ECO_2_). Data are presented according to temperature (*T* or *T* + 4) and CO_2_ regime (ACO_2_ or ECO_2_) and considering each clone individually (values are means ± SE, *n* = 3–4). **(A)** Throughout ripening and **(B)** at maturity. Relative abundance of amino acids **(C)** grouped by their precursor. Means with letters in common are not significantly different according to the LSD test (*P* > 0.05). Probability values (*P*) for the main effects of clone, *P*_(CL)_; temperature, *P*_(_*_*T*_*_)_; and CO_2_, *P*_(CO__2__)_. ***P < 0.001; *P < 0.05; ns, not significant. All probability values for the interactions of factors [*P*_(CL ×_ *_*T*_*_)_, *P*_(CL × *CO*__2__)_, *P*_(_*_*T*_*_× *CO*__2__)_, and *P*_(CL ×_ *_*T*_*_× *CO*__2__)_] were statistically not significant (*P* > 0.05).

In general, total amino acid concentration reached the highest values 2 weeks after mid-veraison in clones RJ43, CL306, and VN31, whereas the concentration of amino acid continued increasing until maturity in T3 and 1084 (Figure 4A and Supplementary Tables 1A–E). Amino acid levels were similar among clones throughout berry development, except 2 weeks after mid-veraison, when 1084 had lower levels in comparison with the other clones. At this stage, and considering all the clones, the *T* + 4 treatment reduced total amino acid concentration compared with the *T* treatment (from 15.9 ± 1.33 to 12.3 ± 1.18 μmol ml^–1^, respectively, Supplementary Table 1D). Also, ECO_2_ diminished the amino acid levels with respect to ACO_2_ (from 17.1 ± 1.48 to 11.2 ± 0.82 μmol ml^–1^, respectively) 2 weeks after mid-veraison (Supplementary Table 1D). At maturity, there were no significant effects of temperature and CO_2_ (neither individually nor combined). However, *T* + 4 tended to reduce the concentration of total amino acids (especially under ACO_2_) in all the clones and ECO_2_ tended to reduce the amino acid levels of CL306, T3, and VN31 at ambient temperature (Figure 4B and Supplementary Table 1E).

The relative abundance of the different amino acids varied among clones. Specifically, 1 and 2 weeks after mid-veraison and at maturity, the pyruvate and aspartate derivatives were more abundant in 1084 at the expense of α-ketoglutarate derivatives (except GABA and arginine, which increased) (Figure 4C and Supplementary Tables 1D,E). Considering all the clones as a whole, ECO_2_ significantly reduced the proportion of α-ketoglutarate derivatives (although arginine and GABA tended to increase in the later stages of the ripening period at ECO_2_), increasing the abundance of those originated from aspartate and pyruvate (2 weeks after mid-veraison and at maturity, respectively). Even though there were no significant interactions, there were two exceptions to this effect at maturity, as ECO_2_ increased α-ketoglutarate derivatives in the grapes of clones RJ43 and 1084 exposed to *T* and *T* + 4, respectively. In addition, the rise in the relative abundance of pyruvate derivatives observed in the ECO_2_ treatment at maturity was globally stronger at *T* + 4 (Figure 4C and Supplementary Table 1E). Moreover, at the onset of veraison, ECO_2_ strongly increased the relative abundance of phenylalanine in all clones and more specifically in CL306 and 1084 when combined with elevated temperature.

### Total Skin Anthocyanins

**FIGURE 5:**
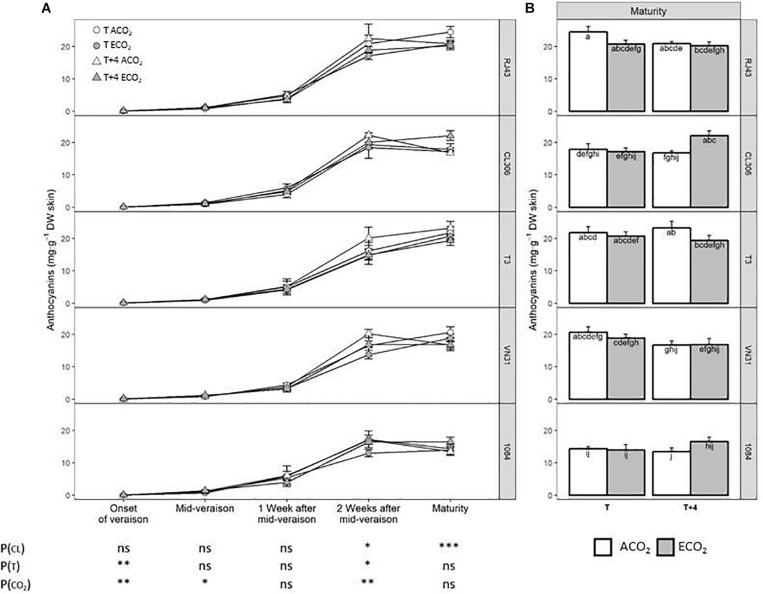
Total anthocyanin concentration in berries of the five Tempranillo clones grown under four temperature/CO_2_ regimes: ambient temperature (*T*) or ambient temperature + 4°C (*T* + 4), combined with ambient CO_2_ (ca. 400 ppm, ACO_2_) or elevated CO_2_ (700 ppm, ECO_2_). Data are presented according to temperature (*T* or *T* + 4) and CO_2_ regime (ACO_2_ or ECO_2_) and considering each clone individually (values are means ± SE, *n* = 4). **(A)** Throughout ripening and **(B)** at maturity. Means with letters in common are not significantly different (*P* > 0.05) according to the LSD test. Probability values (*P*) for the main effects of clone, *P*_(CL)_; temperature, *P*_(_*_*T*_*_)_; and CO_2_, *P*_(CO__2__)_. ***P < 0.001; **P < 0.01; *P < 0.05; ns, not significant. All probability values for the interactions of factors [*P*_(CL ×_ *_*T*_*_)_, *P*_(CL × *CO*__2__)_, *P*_(_*_*T*_*_× *CO*__2__)_, and *P*_(CL ×_ *_*T*_*_× *CO*__2__)_] were statistically not significant (*P* > 0.05).

Anthocyanin levels did not differ significantly in the early ripening period among clones, but 2 weeks after mid-veraison and at maturity, the concentration of total anthocyanins was lower in 1084, whereas RJ43 showed the highest values (Figures 5A,B). *T* + 4 had a significant enhancing effect on anthocyanins at the onset of veraison and 2 weeks after mid-veraison, regardless of the clone and CO_2_ level, this effect disappearing at maturity. ECO_2_ increased anthocyanin concentration at the onset of veraison and mid-veraison but reduced the levels 2 weeks after mid-veraison (Figure 5A). At maturity, although there were no significant interactions between factors, the *T* + 4/ECO_2_ treatment seemed to have different effects depending on the clone. Whereas, in RJ43, the grapes exposed to *T* + 4/ECO_2_ (climate change conditions) had significantly lower anthocyanin levels than those exposed to *T*/ACO_2_ (current conditions), in CL306, the concentration of skin anthocyanins increased with climate change conditions (*T* + 4/ECO_2_) (Figure 5B).

### Total Anthocyanins to TSS Ratio

**FIGURE 6:**
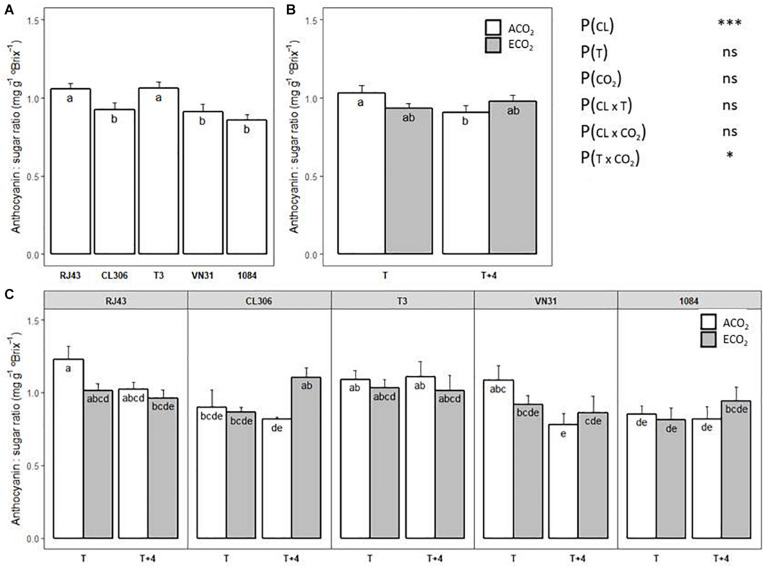
Anthocyanin to TSS ratio at maturity of the five Tempranillo clones grown under four temperature/CO_2_ regimes: ambient temperature (*T*) or ambient temperature + 4°C (*T* + 4), combined with ambient CO_2_ (ca. 400 ppm, ACO_2_) or elevated CO_2_ (700 ppm, ECO_2_). Results (values are means ± SE) are presented according to: **(A)** clone identity (*n* = 15–16); **(B)** the temperature (*T* or *T* + 4) and CO_2_ regime (ACO_2_ or ECO_2_) (*n* = 19–20); and **(C)** clone identity, temperature, and CO_2_ regime (*n* = 3–4). Means with letters in common within each chart **(A–C)** are not significantly different according to the LSD test (*P* > 0.05). Probability values (*P*) for the main effects of clone, *P*_(CL)_; temperature, *P*_(_*_*T*_*_)_; and CO_2_, *P*_(CO__2__)_. ***P < 0.001; *P < 0.05; ns, not significant. All probability values for the interactions of factors [*P*_(CL ×_ *_*T*_*_)_, *P*_(CL × *CO*__2__)_, *P*_(_*_*T*_*_× *CO*__2__)_, and *P*_(CL ×_ *_*T*_*_× *CO*__2__)_] were statistically not significant (*P* > 0.05).

Clones showed different anthocyanin to TSS ratios at maturity regardless of the temperature and CO_2_ regime, RJ43 and T3 having the highest values (Figure 6A). Considering the clones altogether, a significant interaction between temperature and CO_2_ was observed (Figure 6B). Thus, the significant decrease of the ratio between anthocyanins and TSS under ACO_2_ induced by *T* + 4, with respect to *T*, disappeared under ECO_2_. When the temperature and CO_2_ effects were analyzed for each clone independently, the growing conditions showed slightly different effects on the anthocyanin to TSS ratio (Figure 6C). In RJ43 and VN31, the impact of *T* + 4 on the ratio was more evident under ACO_2_ conditions. ECO_2_ strongly increased the ratio in CL306 plants at *T* + 4, while it did not have any effect at *T*. Finally, neither temperature nor CO_2_ had a marked impact on the relationship between anthocyanins and TSS in T3 and 1084.

## Discussion

### Performance of Clones

Clones showed different characteristics in all the studied parameters. The accession that differed the most from the others studied was 1084. It had an extremely long berry ripening period associated with a lower sugar accumulation rate, not even reaching the optimum sugar levels for wine production. The 1084 accession also had berries with low relative skin mass and presented the lowest values of malic acid at maturity. Despite T3, VN31, and RJ43 having similar berry diameter to 1084 (indicating similar size), their malic acid values were higher than in 1084. This result suggests that the low concentration of malic acid in 1084 was not associated with a dilution effect due to high berry size. After veraison, malate is released from the vacuole and becomes available for catabolism through involvement in gluconeogenesis, respiration through the tricarboxylic acid (TCA) cycle, amino acid interconversions, and the production of secondary compounds such as anthocyanins and flavonols (Ruffner and Kliewer, 1975; Ruffner et al., 1976; Ruffner, 1982; Famiani et al., 2000; Ollat et al., 2002; Sweetman et al., 2009). Probably, the longer ripening period of the 1084 accession, already seen in previous experiments (Arrizabalaga et al., 2018), contributed to higher malate breakdown, thus reaching a lower malic acid concentration at maturity. Interestingly, clones differed in the moment to reach the amino acidic maturity, earlier in RJ43, CL306, and VN31 than in T3 and 1084. The amino acid profile at maturity was also different among clones, 1084 showing a reduced proportion of α-ketoglutarate and increased abundance of pyruvate and aspartate derivatives. The higher concentration of isoleucine, leucine, and valine (aromatic precursors) in 1084 might have a positive effect on the final wine aroma (Valdés et al., 2019).

The 1084 accession also presented a lower concentration of skin anthocyanins, compared with the other clones. The results are in agreement with our previous work (Arrizabalaga et al., 2018) and may be related to the slow sugar accumulation rate observed in 1084. Dai et al. (2014), using an experimental system allowing the long-term *in vitro* culture of grape berries, reported an induction in total anthocyanins by rising sugar concentrations in the culture medium, as well as a negative correlation between phenylalanine and total anthocyanin levels. In the present study, phenylalanine was similar in 1084, and even higher 2 weeks after mid-veraison, compared with the other clones. Therefore, the lower anthocyanin accumulation may be a consequence of a limitation in the biosynthetic enzymatic activity rather than to a limitation of its precursor, as suggested by Dai et al. (2014). Also, it is possible that α-ketoglutarate availability is lower in 1084; thus, biosynthetic steps that use α-ketoglutarate as reducing power for anthocyanin accumulation (i.e., hydroxylases) become limiting. Indeed, α-ketoglutarate level was reported to be one of the metabolic drivers of anthocyanin accumulation in grape cells (Soubeyrand et al., 2018). In opposition to the results obtained by Roby and Matthews (2008), the anthocyanin content in berries was not systematically positively correlated to the relative skin mass, except for 1084. These discrepancies may be related to the fact that in our case anthocyanins were measured in dry skin and Roby and Matthews (2008) did it in the whole berry. In addition, both 1084 and T3 had some of the largest diameters. However, the relative skin mass of this two clones differed significantly, T3 having a higher value and 1084 the lowest. These results indicate a lack of effect of berry size on the relative skin mass already observed by Barbagallo et al. (2011). These berry parameters are important as they are considered to determine the solutes extraction during the maceration process (Walker et al., 2005; Matthews and Nuzzo, 2007; Roby and Matthews, 2008; Barbagallo et al., 2011).

### Global Response of Tempranillo Clones to Elevated Temperature

The increment of temperature shortened the elapsed time between mid-veraison and maturity and reduced the size of berries at the end of the ripening process. These results agree with previous studies, in which berries stopped increasing their volume after being heat-treated at mid-veraison and mid-ripening (Kliewer, 1977; Roby and Matthews, 2008; Keller, 2010; Orduña, 2010) and high temperature accelerated the ripening process (Jones and Davis, 2000; Duchêne and Schneider, 2005; Keller, 2010; Arrizabalaga et al., 2018). Given that maturity was determined by the level of TSS (ca. 22°Brix), the reduction of the time to reach this stage in the *T* + 4 treatment indicates a faster and more efficient accumulation of sugars under these conditions. In the present study, the lower berry size in the *T* + 4 may have contributed to the higher concentration of sugars observed. However, the effect of high temperatures on sugar accumulation varies among studies and conditions. Whereas elevated temperature has been reported to enhance sugar accumulation (Jones et al., 2005; Mosedale et al., 2016), in other cases, berry sugar content at harvest was not affected (Lecourieux et al., 2017), and sugar accumulation was stopped (Roby and Matthews, 2008; Greer and Weston, 2010), or even decreased (Carbonell-Bejerano et al., 2013; Greer and Weedon, 2013; Kuhn et al., 2014). These apparently contradictory results may be due to differences in the experimental procedures, which included more extreme temperatures than in the present one, thus reducing photosynthesis and limiting the supply of sugar to the berries (Greer and Weedon, 2013).

It is well established that warm temperatures promote the decrease of organic acid levels in grape berries after mid-veraison, by accelerating malate degradation (Orduña, 2010; Carbonell-Bejerano et al., 2013; Sweetman et al., 2014; Torres et al., 2016). Malic acid respiration is favored by heat, and the genes involved in its transmembrane transport display a marked regulation by temperature (Rienth et al., 2016). In addition, the enhancement of the anaplerotic capacity of the TCA cycle for amino acid biosynthesis by elevated temperatures has also been suggested (Sweetman et al., 2014; Lecourieux et al., 2017). In our work, although malic acid degradation was promoted by *T* + 4 from mid-veraison onward, the concentration of total amino acids tended to decrease in this treatment compared with *T*, and changes in the proportion α-ketoglutarate, pyruvate, or aspartate amino acid derivatives were not so obvious. These results may indicate that under the temperature conditions assayed, 4°C of difference between *T* and *T* + 4 vs. increases of 8 (Lecourieux et al., 2017) and 10°C (Sweetman et al., 2014) in the mentioned studies, the anaplerotic capacity of the TCA cycle for amino acid biosynthesis may not be markedly increased. Other pathways such as gluconeogenesis may have played a more important role in malate degradation, thus contributing to the differences in sugar accumulation observed between temperature regimes. Despite the limited changes in the amino acid profile, an increased proportion of proline and arginine was observed under high temperature, as previously reported by other authors (Lecourieux et al., 2017; Torres et al., 2017). Proline has a protective role in plants against abiotic stresses, including elevated temperature, whereas arginine is an important source of nitrogen during winemaking (Garde-Cerdán et al., 2011), despite being a precursor of putrescine, a compound with negative effects on human health (Guo et al., 2015).

Considering the clones altogether, high temperature significantly increased the concentration of anthocyanins 2 weeks after mid-veraison, but it did not affect the final anthocyanin levels at maturity. These results do not agree with previous studies that demonstrate that high temperature during ripening had a negative impact on anthocyanin biosynthesis in berries by acting on the correspondent enzymes and transcription factors (Yamane et al., 2006; Mori et al., 2007; Rienth et al., 2014, 2016; Lecourieux et al., 2017). In those experiments, the expression of genes related to flavonoid biosynthesis in grape skins was found to be repressed by high temperatures, especially genes coding for the key enzyme of the phenylpropanoid pathway, the phenylalanine-ammonia-lyase (PAL) or *MYB* transcription factors, which control anthocyanin biosynthesis. However, the repression of *VvMYBA1* by high temperature described by Yamane et al. (2006) was not confirmed by other authors (Lijavetzky et al., 2012). Besides, in addition to a lower anthocyanin biosynthesis, some authors have reported increases in anthocyanin degradation due to high temperature (Mori et al., 2007). Accordingly, the increase in anthocyanin concentration induced by *T* + 4 observed 2 weeks after mid-veraison in our study was not detected at maturity (*P*_(_*_*T*_*_)_ > 0.05), which might be caused by an earlier degradation of anthocyanins under elevated temperature.

Anthocyanin levels have also been reported to be differentially affected by temperature in different cultivars (Downey et al., 2006). Total anthocyanins decreased with high temperature in Merlot (Spayd et al., 2002; Tarara et al., 2008), Malbec (de Rosas et al., 2017), Pione (Azuma et al., 2012), Cabernet Sauvignon (Mori et al., 2007; Lecourieux et al., 2017; Wu et al., 2019), and Muscat Hamburg (Carbonell-Bejerano et al., 2013), while the final concentration of anthocyanins increased in Merlot by reducing the day temperature oscillations (Cohen et al., 2008). In the case of Tempranillo, Kizildeniz et al. (2018), in a 3-year experiment under similar conditions to this study, did not observe significant effects of high temperature on final anthocyanin levels. Also, differences in the response of anthocyanins to temperature were detected among different clones of Tempranillo, not all the clones being equally affected (Torres et al., 2017; Arrizabalaga et al., 2018). Furthermore, some authors also reported that the stage of berry growth at which heat treatment was applied could modulate the plant response in terms of anthocyanin content (Lecourieux et al., 2017; Gouot et al., 2019a, b).

### Global Response of Tempranillo Clones to Elevated CO_2_

High atmospheric CO_2_ concentration slightly hastened grape phenology, but only from fruit set to mid-veraison, as reported by Martínez-Lüscher et al. (2016a) for red and white Tempranillo. Regarding fruit composition, the increase in malic acid concentration of grapes exposed to ECO_2_ at the onset of veraison may indicate an enhancement of the organic acid biosynthesis at early berry development stages. These differences disappeared in later stages, when the degradation of malic acid took place, and the grapes ripened under ECO_2_ conditions reaching even lower malic acid levels at maturity than those grown at ACO_2_, possibly because of an accelerated breakdown. Similarly, Bindi et al. (2001), in a field experiment using a free air CO_2_ enrichment (FACE) facility, observed that organic acid components were positively affected by increases in CO_2_ concentration at the middle of the ripening season, an effect that almost completely vanished at maturity. On the other hand, ECO_2_ significantly decreased the amino acid concentration at the onset of veraison and 2 weeks after mid-veraison. Elevated CO_2_ may induce a priority in the storage of nitrogen-free compounds (i.e., carbohydrates) compared with nitrogen-containing compounds, such as amino acids and proteins (Myers et al., 2014). In our case, the concentration of sugars in the berries was not significantly altered by ECO_2_ at these stages, so the decrease in amino acids could be a consequence of the inhibition of N assimilation as reported in different plant species (Serret et al., 2018). Similarly, in a previous study with the same clones and under similar growth conditions, berries exposed to ECO_2_ showed lower N concentration in the berries (Arrizabalaga-Arriazu et al., 2020). The differences observed up to 2 weeks after veraison in the present study decreased at maturity, when the ECO_2_ treatment showed only a slightly lower total amino acid concentration. At this stage, even though the relative abundance of some individual amino acids was significantly affected by ECO_2_ conditions, the proportions of amino acid families according to their precursor were little modified, except a slightly reduced proportion of α-ketoglutarate derivatives, at the expense of those originated from pyruvate. The significant decrease in the concentration of glutamine under ECO_2_ may involve a limitation for yeast growth during fermentation since glutamine, together with arginine, is one of the mayor sources of N for yeast (Zoecklein et al., 1999).

Taking into consideration the clones altogether, plants grown at ECO_2_ presented an increased concentration of total grape skin anthocyanins at the beginning of the ripening period, which may indicate a slight hastening in the synthesis of these compounds in this treatment. In contrast, 2 weeks after mid-veraison, ECO_2_ plants had lower anthocyanin concentration than ACO_2_, these differences disappearing at maturity. These results do not agree with the observations of other authors, who reported no effect (Gonçalves et al., 2009) or a rising effect of CO_2_ on anthocyanins at maturity (Kizildeniz et al., 2015). In the same way, studies on the effects of elevated CO_2_ on the anthocyanin biosynthetic pathway in different plant species report contradictory results. For example, in strawberry and table grapes, elevated CO_2_ levels decreased anthocyanin content by decreasing the expression of genes involved in the phenylpropanoid metabolism, especially the one coding the PAL enzyme (Sanchez-Ballesta et al., 2007; Li et al., 2019). In contrast, increased anthocyanin content and stimulated enzyme activity have been observed in ginger and *Labisia pumila* under elevated CO_2_ (Ghasemzadeh et al., 2012; Jaafar et al., 2012).

### Response of Clones to Combined Elevated Temperature and CO_2_

The analysis of the combined effects of high temperature and elevated CO_2_ on the parameters analyzed indicates low interactive effects of these environmental factors on sugar accumulation and, thus, in the length of the ripening period, as ECO_2_ did not modify the hastening effect induced by *T* + 4. Conversely, the increase in temperature and CO_2_ showed additive effects enhancing malic acid degradation, a phenomenon especially marked in the 1084 accession (probably due to its longer exposure to these conditions), but less evident in RJ43 and CL306. Such reduction of malic acid content and its impact on must acidity should certainly affect wine production, not only for its contribution to sourness and organoleptic properties but also because of its influence on wine microbiological stability. In addition, it might make the winemaking process more expensive in the future due to the need of acidifying the must for achieving a proper fermentation (Keller, 2010). The combined application of elevated temperature and high CO_2_ did not seem to have a marked impact on the total level of amino acids at maturity, although a tendency to decrease the amino acid concentrations was observed as reported by Martínez-Lüscher et al. (2016a). This effect was more evident 2 weeks after mid-veraison, *T* + 4 and ECO_2_ significantly reducing their concentration. These results suggest an impact of climate change delaying the amino acidic maturity of grapes, which seemed to be reached some weeks earlier in the *T*/ACO_2_ treatment. Amino acids (excluding proline and hydroxyproline) are very important components of the yeast assimilable nitrogen (YAN), thus having implication for must fermentation. A decrease of total amino acids level could involve a lower N availability during the fermentation process. In the same way, a reduction in the concentration of shikimate and phosphoglycerate derivatives may have implications in the organoleptic properties of the wine produced from these grapes, since the shikimate route is involved in the biosynthesis of aromatic amino acids (tyrosine and phenylalanine) and the phenylpropanoid pathway (phenylalanine).

As for amino acids, combined elevated temperature and CO_2_ did not significantly impact anthocyanin concentration at maturity when considering all the clones as a whole. However, the observed increase due to high temperature 2 weeks after veraison was offset by combination with elevated CO_2_. Interestingly, a significant interaction between temperature and CO_2_ was observed for the relationship between anthocyanin and sugar levels: the temperature-induced decrease in this ratio under ACO_2_ was not observed under ECO_2_. This result suggests that in a future climate change scenario, elevated CO_2_ may, at least partially, mitigate the negative impact of high temperature on the imbalance between sugars and anthocyanins in ripe berries.

In addition, regarding the anthocyanin content, there was a wide range of responses of the clones studied to climate change conditions. Total anthocyanin concentration at maturity was impacted by combined elevated temperature and CO_2_, showing a decrease in RJ43, T3, and VN31 (stronger in the former), despite their difference in the reproductive cycle length (defined as intermediate, short, and long, respectively). Conversely, combined elevated temperature and CO_2_ increased anthocyanin content in CL306 (characterized as a short reproductive cycle clone) and in 1084 (long reproductive cycle). Moreover, while in RJ43 and VN31 climate change conditions (*T* + 4/ECO_2_) markedly reduced the anthocyanin to sugar ratio with respect to the current conditions (*T*/ACO_2_) (differences statistically significant in RJ43), the balance between these two compounds was less affected in CL306, T3, and 1084. Grapevine is characterized by a pronounced sensitivity toward the environment, and the metabolic composition of the berries has been reported to show a broad phenotypic plasticity, offering advantages such as the range of different wines that can be produced from the same cultivar and the adaptation of existing cultivars to different growing regions (Keller, 2010). The present results suggest that clones of the same variety may perform differently under climate change conditions, some, such as RJ43, showing greater variation and others, such as 1084, offering more consistency. Unfortunately, we cannot directly associate these different performances with the length of their grape development period as we hypothesized originally, at least as far as anthocyanins and anthocyanin to sugar ratio are concerned. In this line, although 1084, with the longest length of the reproductive cycle, was one of the least affected in terms of anthocyanin and sugar balance, other clones that exhibited an early maturity (CL306 and T3) were not so affected either. A recent study has demonstrated differences in the berry phenotypic plasticity between grapevine varieties in response to changes in environmental conditions, Cabernet Sauvignon being less dependent on growth conditions, thus showing a limited transcriptomic plasticity associated with epigenetic regulation (Dal Santo et al., 2018). These authors also concluded that within-cultivar diversity may modulate gene expression in response to environmental cues. We suggest that clones within the same variety can also exhibit different degrees of phenotypic plasticity for grape composition and, consequently, different capacities of adaptation to climate change conditions. Recently, Tortosa et al. (2019) have reported that some clones of Tempranillo variety can perform better water use efficiency than others depending of the water availability conditions. Although the results obtained need to be considered in the light of the limitations of the study (i.e., 1 year experiment, potted plants, and controlled conditions), and they should be validated with studies in the field, the differences observed point toward the usefulness of the exploitation of the grapevine genotypic diversity in order to optimize the genotype–environment interaction and the adaptation of traditional varieties to the foreseen climate scenarios.

## Conclusion

The projected increases in atmospheric CO_2_ concentration and in average air temperature advanced grape maturity, reducing the elapsed time between fruit set and mid-veraison and between mid-veraison and maturity, respectively. High temperature hastened berry ripening, sugar accumulation, and malic acid breakdown, especially when combined with elevated CO_2_. In contrast, climate change conditions seemed to delay amino acidic maturity. Even though the increase of temperature and high CO_2_ concentration (both individually and combined) did not affect anthocyanin concentration, the clones studied showed different values for this parameter. The reduction of the ratio between anthocyanins and TSS under *T* + 4 conditions was partially mitigated by ECO_2_. Additionally, the study reveals the existence of a differential response of Tempranillo clones to the projected future temperature and CO_2_ levels in terms of grape composition.

## Data Availability Statement

The raw data supporting the conclusions of this article will be made available by the authors, without undue reservation.

## Author Contributions

FM, GH, IP, and JI: conceptualization. GH and IP: methodology. MA-A: investigation. JI: resources, project administration, and funding acquisition. GH, IP, and MA-A: data curation and writing – original draft preparation. EG, FM, GH, IP, JI, and MA-A: writing – review and editing. GH, IP, and JI: supervision. All authors approved the manuscript for publication.

## Conflict of Interest

The authors declare that the research was conducted in the absence of any commercial or financial relationships that could be construed as a potential conflict of interest.

## Supplementary Material

Supplementary Figure 1 Daily minimum and maximum temperatures recorded in the modules of ambient temperature (T) and ambient temperature + 4°C (T + 4) of the TGGs during the experiment.

Supplementary Figure 2 Total soluble solids concentration at maturity in berries of the five Tempranillo clones grown under four temperature/CO_2_ regimes: ambient temperature (T) or ambient temperature + 4°C (T + 4), combined with ambient CO_2_ (ca. 400 ppm, ACO_2_) or elevated CO_2_ (700 ppm, ECO_2_). Data are presented according to the temperature (T or T + 4) and CO_2_ regime (ACO_2_ or ECO_2_) and considering each clone individually (values are means ± SE, *n* = 4). Means with letters in common are not significantly different according to LSD test (*P* > 0.05).
