# Supplementary material for: High Temperature and Elevated Carbon Dioxide Modify Berry Composition of Different Clones of Grapevine (Vitis vinifera L.) cv. Tempranillo
Source: Front Plant Sci. 2020 Dec 1;11:603687. doi: 10.3389/fpls.2020.603687 (PMC7736076; doi:10.3389/fpls.2020.603687)
Supplement: Supplementary Figure 1 — Daily minimum and maximum temperatures recorded in the modules of ambient temperature (T) and ambient temperature + 4°C (T + 4) of the TGGs during the experiment. [file Image_1.pdf]

Supporting informations

High temperature and elevated CO_2 modify berry composition of different clones of grapevine (*Vitis vinifera* L.) cv. Tempranillo

Marta Arrizabalaga-Arriazu[1,2], Eric Gomès[2], Fermín Morales[3], Juan José Irigoyen[1], Inmaculada Pascual[1], Ghislaine Hilbert[2*]

[1]Universidad de Navarra. Faculty of Sciences. Plant Stress Physiology Group, Associated Unit to CSIC (EEAD, Zaragoza, and ICVV, Logroño). Irunlarrea, 1. 31008, Pamplona, Spain.

[2]EGFV, Univ. Bordeaux, Bordeaux Sciences Agro, INRAE, ISVV, F-33882, Villenave d'Ornon, France

[3]Instituto de Agrobiotecnología (IdAB), Consejo Superior de Investigaciones Científicas (CSIC)- Gobierno de Navarra, Avenida Pamplona 123, 31192, Mutilva, Spain.

*** Correspondence:**

Ghislaine Hilbert

EGFV, Univ. Bordeaux, Bordeaux Sciences Agro, INRAE, ISVV, F-33882, Villenave d'Ornon, France

ghislaine.hilbert@inrae.fr

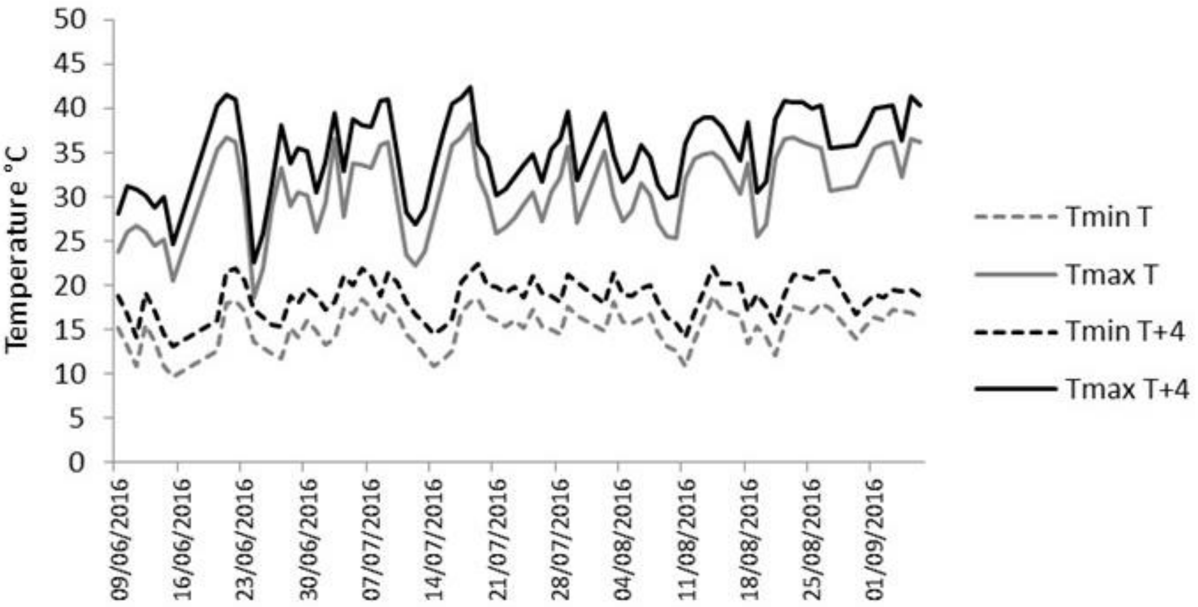

Figure S1| Daily minimum and maximum temperatures recorded in the modules of ambient temperature (T) and ambient temperature +4 °C (T+4) of the TGGs during the experiment.

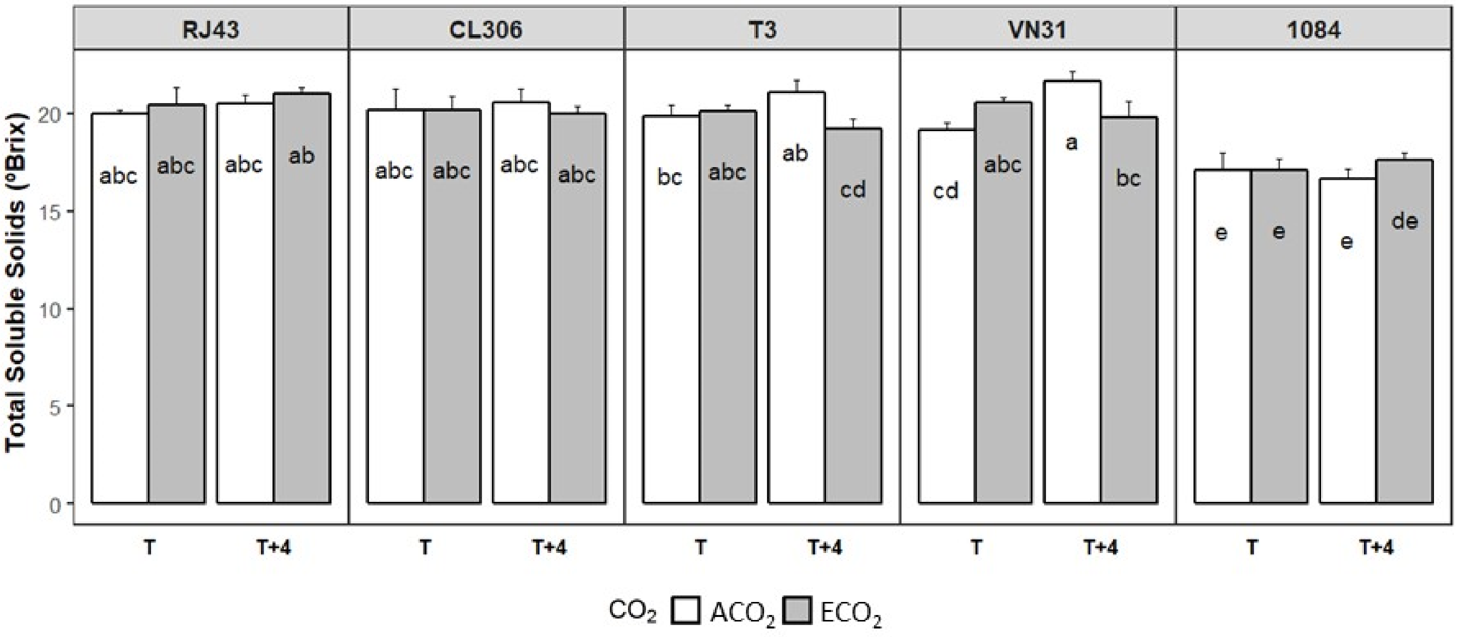

Figure S2| Total soluble solids concentration at maturity in berries of the five Tempranillo clones grown under four temperature/CO2 regimes: ambient temperature (T) or ambient temperature + 4 °C (T+4), combined with ambient CO2 (ca. 400 ppm, ACO2) or elevated CO2 (700 ppm, ECO2). Data are presented according to the temperature (T or T+4) and CO2 regime (ACO2 or ECO2) and considering each clone individually (values are means ± SE, n=4). Means with letters in common are not significantly different according to LSD test (P > 0.05).
