# Supplementary material for: High Temperature and Elevated Carbon Dioxide Modify Berry Composition of Different Clones of Grapevine (Vitis vinifera L.) cv. Tempranillo
Source: Front Plant Sci. 2020 Dec 1;11:603687. doi: 10.3389/fpls.2020.603687 (PMC7736076; doi:10.3389/fpls.2020.603687)
Supplement: Supplementary file 2 [file Table_1.DOCX]

Table S1A. Total free amino acid content (µmol mL^-1^) and relative abundance of individual amino acids (%) at the onset of veraison in berries of the five Tempranillo clones grown under four temperature/CO_2_ regimes: ambient temperature (T) or ambient temperature + 4 °C (T+4), combined with ambient CO_2_ (ca. 400 ppm, ACO_2_) or elevated CO_2_ (700 ppm, ECO_2_). Probability values (P) for the main effects of clone, P(CL); temperature P(T); CO_2_, P(CO_2_). ***, P < 0.001; **, P < 0.01; *, P < 0.05; ns, not significant. All probability values for the interactions of factors (P(CL x T), P(CL x CO_2_), P(T x CO_2_) and P(CL x T x CO_2_)) were statistically not significant (P > 0.05).

Table S1B. Total free amino acid content (µmol mL^-1^) and relative abundance of individual amino acids (%) at mid-veraison in berries of the five Tempranillo clones grown under four temperature/CO_2_ regimes: ambient temperature (T) or ambient temperature + 4 °C (T+4), combined with ambient CO_2_ (ca. 400 ppm, ACO_2_) or elevated CO_2_ (700 ppm, ECO_2_). Probability values (P) for the main effects of clone, P(CL); temperature P(T); CO_2_, P(CO_2_); and their interactions, P(CL x T), P(CL x CO_2_), P(T x CO_2_) and P(CL x T x CO_2_).***, P < 0.001; **, P < 0.01; *, P < 0.05; ns, not significant.

Table S1C. Total free amino acid content (µmol mL^-1^) and relative abundance of individual amino acids (%) one week after mid-veraison in berries of the five Tempranillo clones grown under four temperature/CO_2_ regimes: ambient temperature (T) or ambient temperature + 4 °C (T+4), combined with ambient CO_2_ (ca. 400 ppm, ACO_2_) or elevated CO_2_ (700 ppm, ECO_2_). Probability values (P) for the main effects of clone, P(CL); temperature P(T); CO_2_, P(CO_2_); and their interactions, P(CL x T), P(CL x CO_2_) and P(T x CO_2_). ***, P < 0.001; **, P < 0.01; *, P < 0.05; ns, not significant. All probability values for the interaction P(CL x T x CO_2_) were statistically not significant (P > 0.05).

Table S1D. Concentration of total free amino acid (µmol mL^-1^) and relative abundance of individual amino acids (%) two weeks after mid-veraison of the five Tempranillo clones grown under four temperature/CO_2_ regimes: ambient temperature (T) or ambient temperature + 4 °C (T+4), combined with ambient CO_2_ (ca. 400 ppm, ACO_2_) or elevated CO_2_ (700 ppm, ECO_2_). Probability values (P) for the main effects of clone, P(CL); temperature P(T); CO_2_, P(CO_2_); and their interactions, P(CL x T), P(CL x CO_2_) and P(T x CO_2_). ***, P < 0.001; **, P < 0.01; *, P < 0.05; ns, not significant. All probability values for the interaction P(CL x T x CO_2_) were statistically not significant (P > 0.05).

Table S1E. Total free amino acid content (µmol mL^-1^) and relative abundance of individual amino acids (%) at maturity in berries of the five Tempranillo clones grown under four temperature/CO_2_ regimes: ambient temperature (T) or ambient temperature + 4 °C (T+4), combined with ambient CO_2_ (ca. 400 ppm, ACO_2_) or elevated CO_2_ (700 ppm, ECO_2_). Probability values (P) for the main effects of clone, P(CL); temperature P(T); CO_2_, P(CO_2_); and their interactions, P(CL x T), P(CL x CO_2_) and P(T x CO_2_). ***, P < 0.001; **, P < 0.01; *, P < 0.05; ns, not significant. All probability values for the interaction P(CL x T x CO_2_) were statistically not significant (P > 0.05).
